# Associations between meteorological parameters and influenza activity in a subtropical country: Case of five sentinel sites in Yaoundé-Cameroon

**DOI:** 10.1371/journal.pone.0186914

**Published:** 2017-10-31

**Authors:** Gwladys C. Monamele, Marie-Astrid Vernet, Robert F. J. Nsaibirni, Jean Joel R. Bigna, Sebastien Kenmoe, Mohamadou Ripa Njankouo, Richard Njouom

**Affiliations:** 1 National Influenza Centre, Centre Pasteur du Cameroun, Yaoundé, Cameroon; 2 Department of Microbiology and Parasitology, University of Buea, Buea, Cameroon; Columbia University, UNITED STATES

## Abstract

Influenza is associated with highly contagious respiratory infections. Previous research has found that influenza transmission is often associated with climate variables especially in temperate regions. This study was performed in order to fill the gap of knowledge regarding the relationship between incidence of influenza and three meteorological parameters (temperature, rainfall and humidity) in a tropical setting. This was a retrospective study performed in Yaoundé-Cameroon from January 2009 to November 2015. Weekly proportions of confirmed influenza cases from five sentinel sites were considered as dependent variables, whereas weekly values of mean temperature, average relative humidity and accumulated rainfall were considered as independent variables. A univariate linear regression model was used in determining associations between influenza activity and weather covariates. A time-series method was used to predict on future values of influenza activity. The data was divided into 2 parts; the first 71 months were used to calibrate the model, and the last 12 months to test for prediction. Overall, there were 1173 confirmed infections with influenza virus. Linear regression analysis showed that there was no statistically significant association observed between influenza activity and weather variables. Very weak relationships (-0.1 < r < 0.1) were observed. Three prediction models were obtained for the different viral types (overall positive, Influenza A and Influenza B). Model 1 (overall influenza) and model 2 (influenza A) fitted well during the estimation period; however, they did not succeed to make good forecasts for predictions. Accumulated rainfall was the only external covariate that enabled good fit of both models. Based on the stationary R^2^, 29.5% and 41.1% of the variation in the series can be explained by model 1 and 2, respectively. This study laid more emphasis on the fact that influenza in Cameroon is characterized by year-round activity. The meteorological variables selected in this study did not enable good forecast of future influenza activity and certainly acted as proxies to other factors not considered, such as, UV radiation, absolute humidity, air quality and wind.

## Introduction

Influenza is associated with highly contagious respiratory illness throughout the world. Illnesses can result in hospitalization and death mainly among high-risk groups (the very young, elderly or chronically ill). Annually, epidemics due to influenza result in approximately 3 to 5 million cases of severe illness, and about 250 000 to 500 000 deaths [1]. The resulting economic impact is large and includes both direct and indirect cost [2–4].

There are different patterns in the circulation of human influenza in tropical and subtropical regions [5]. All countries with winter seasons have a clear peak in influenza activity during the winter months [6]. However, in countries with tropical or subtropical climates, there is no distinct profile in the circulation of influenza, year-round activity can be observed with higher frequency during the rainy seasons [6]. Despite recognition of this phenomenon, mechanisms driving influenza seasonality are not well understood.

Several factors have been found to favour transmission of influenza virus transmission. These factors act through different mechanisms by providing appropriate conditions for: influenza virus survival and increased contact with infected individuals especially in circumstances where there is limited immunization in the population to provide herd immunity [7–9]. In this regard, the likelihood of an infection occurring can be affected by meteorological and environmental conditions [9]. Several laboratory-based studies regarding the role of environmental factors on the spread of influenza have been performed particularly associated to humidity and temperature [8, 10]. When the air becomes dry, ambient humidity decreases, causing respiratory particles to evaporate moderately. The lighter drops of respiratory particles can remain in the air for prolonged periods of time [8, 11]. With respect to temperature, Lowen et *al*. showed that transmission of influenza virus decreases with increasing temperature, then reaches a point of complete halt at 30°C [10]. Rainfall is another meteorological parameter that has been thought to be implicated in influenza transmission [12]. The probable hypothesis is that rainfall promotes indoor crowding and human-human contact leading to more contact or aerosol transmission [13].

There is paucity of data on the association between influenza and weather parameters in the tropics [14, 15], and to date, there has been no data reporting associations between meteorological variables and influenza activity in Cameroon. In 2010, Tchidjou et *al*. reported that rain, high relative humidity and low temperature were associated with increased frequency of acute respiratory infections in Cameroon [16]. Another study performed in Cameroon laid emphasis on the seasonality of influenza to be characterized by year-round circulation and semi-annual peaks in incidence [17]. This study was performed in order to fill the gap of knowledge regarding influenza seasonality. Furthermore, it is essential to understand the role of meteorological factors associated with influenza seasonality because it can help in making projections on the dynamics of infection with fluctuations in climatic factors.

## Materials and methods

### Study population and design

Located at the bottom of the Gulf of Guinea, Cameroon has 21,657,488 inhabitants and an area of 475,442 km^2^. Its capital, Yaoundé, has about 4 million inhabitants [18]. The climatic condition in Cameroon alters with altitude and locations. Yaoundé, which is located in the Centre region is endowed with 3° 52' N Latitude and 11° 32' E Longitude. The central region has an equatorial climate with moderate rainfall between March and June and intense precipitation between September and November.

**Fig 1:**
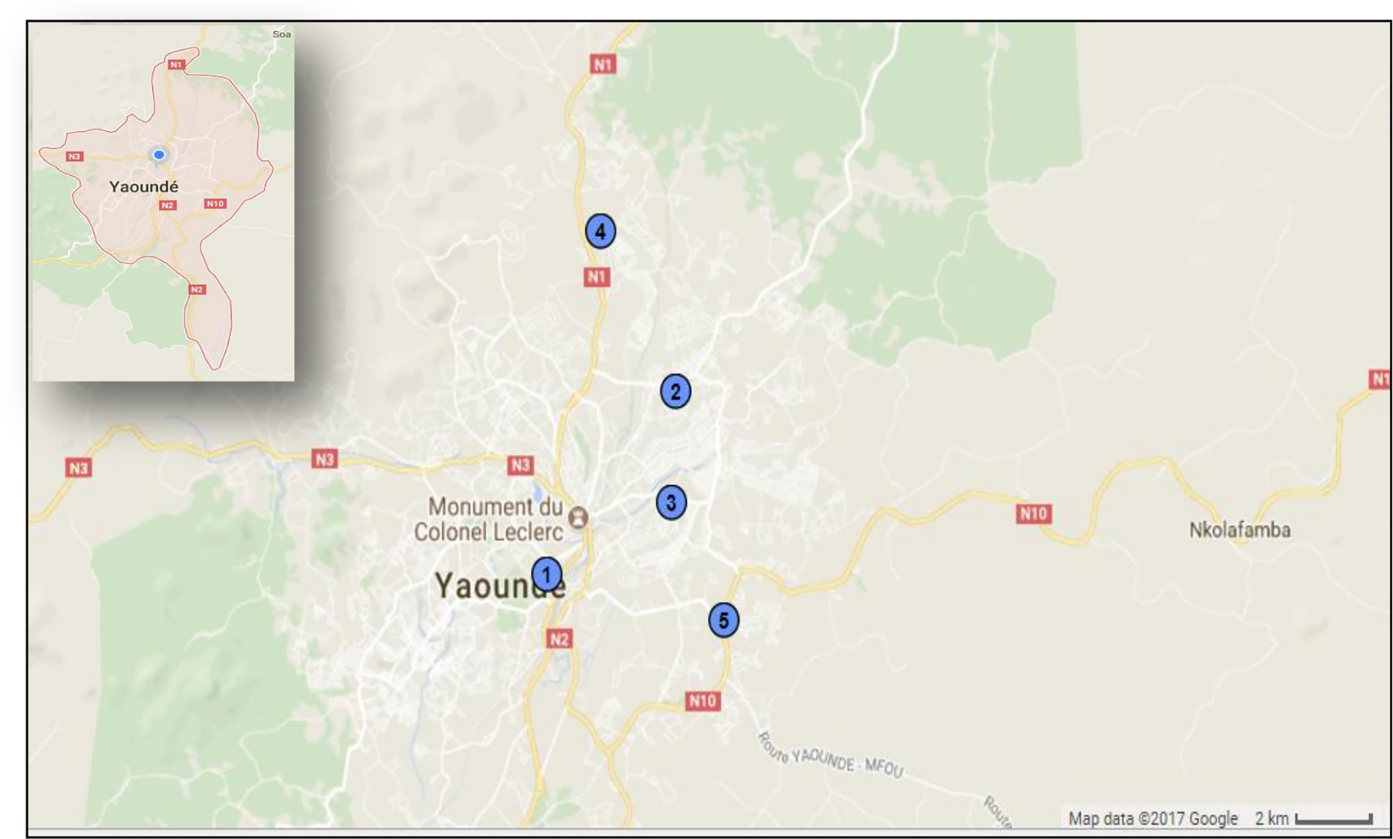
Map showing location of sentinel sites. 1 = “Centre Médico-Social Ambassade de France” (3.85N, 11.51E), 2 = “Centre Hospitalier d’Essos” (3.87N, 11.53E), 3 = “Centre d'Animation Sociale et Sanitaire de Nkolndongo” (3.86N, 11.53E), 4 = “Centre Médical d’Etoudi” (3.92N, 11.52E), 5 = “Centre Médical d’Arrondissement de Nkomo” (3.84N, 11.55E).

This was a retrospective study performed in Yaoundé from January 2009 to November 2015. The study population comprised individuals with Influenza-like illness (ILI) or severe acute respiratory infection (SARI) based on the WHO criteria present in five sentinel clinics in Yaoundé. Nasopharyngeal swab was collected from all individuals that respected the WHO case definition for ILI and SARI throughout the study period. An ILI case was defined as an outpatient presenting sudden onset of fever (temperature >38°C), and cough or sore throat, with the onset of symptoms within the previous 5 days. For SARI, the case definition was similar to that of ILI and in addition required hospital admission. Of the five sentinel sites considered, one received both inpatients and outpatients (“Centre Hospitalier d’Essos”) whereas the four others received only outpatients (“Centre Médico-Social Ambassade de France”, “Centre Médical d’Arrondissement de Nkomo”, “Centre d'Animation Sociale et Sanitaire de Nkolndongo”, “Centre Médical d’Etoudi”). These sites were chosen based on their location (Yaoundé), the completeness of their influenza time series data, and the availability of meteorological parameters for this region. Fig 1 shows the location of the study sites.

### Virological data

Naso-pharyngeal swabs were tested for influenza by real-time reverse transcription polymerase chain reaction (rRT-PCR) using the CDC protocol prior to RNA extraction with the QIAamp Viral RNA Mini Kit (QIAGEN, Hilden, Germany). RNA extraction was performed following the manufacturer’s instructions. These analyses were performed at the National Influenza Centre of Cameroon located in Centre Pasteur of Cameroon which performs influenza surveillance since November 2007 and reports to the World Health Organization (WHO) Global Influenza Surveillance and Response System. All samples were selected in the flu database based on the date of onset of symptoms from January 2009 to November 2015.

### Meteorological data

Meteorological data considered for this study were: relative humidity, temperature and precipitation. These parameters were chosen because they have been reported in several studies to be implicated in influenza transmission [9, 13, 19]. This data was retrieved from the Department of Meteorology at the Ministry of Transport in Cameroon from two different sources. From 2009–2013, meteorological data were obtained from a ground station located at latitude: 3°50'N, longitude: 11°31'E and 760M altitude. Data from 2014 to 2015 were obtained from an automatic station located at latitude 3°8421'N, longitude 11°5242'E and 754M altitude, which records data at a frequency of 15 minutes. The automatic station gives more reliable results but was installed only in 2014. Data obtained from these two sources are specific to Yaoundé, where the five sentinel sites considered in this study are located.

### Analysis

Influenza data was retrieved from the flu database (Access) as an Excel sheet. Individual data for influenza diagnosis was aggregated into daily counts then merged with meteorological data using R version 3.2. This data was further aggregated by week. Weekly proportions of positive laboratory-confirmed cases of influenza A and/or B were considered as dependent variables. As independent variables, weekly values of mean temperature, average relative humidity, and accumulated rainfall were included in the analysis.

A univariate linear regression model was used in determining associations between the different influenza groups (overall influenza, influenza A and influenza B) and weather covariates.

A time series method was as well applied to investigate associations between influenza and weather variability. The time-series data was divided into 2 parts; the first 71 months of data (from January 2009 to November 2014) were used to calibrate the time-series model, and the last 12 months of data (December 2014 to November 2015) were used to test the model prediction. In this study, we used the Statistical Package for Social Sciences (SPSS) Expert Modeller [20, 21], which makes use of the autoregressive integrated moving average model, ARIMA (p,d,q), where p represents the autoregressive (AR) order, d stands for differencing order and q for moving average (MA) order. Autoregressive orders specify which previous values from the series are used to predict current values. Differencing is necessary when trends are present and is used to remove their effect. Moving average orders specify how deviations from the series mean for previous values are used to predict current values. The Ljung–Box (modified Box–Pierce) test was used to determine if the model was correctly specified.

The ARIMA model generally can be written as:

$$Zt+\sum_{p=1}^{P}\emptyset p\;Zt-p=\mu +\sum_{i=1}^{I}\sum_{n=1}^{N}\beta i,\;n\;Xi,\;t-n+\sum_{q=1}^{Q}\theta q\;\varepsilon t-q$$

Where, *Z*_*t*_ is the influenza count at week t, *Z*_*t-p*_ is the count at previous p week (AR), *μ* is the intercept, *X*_*i*,*t-n*_ is environmental variable i with lag n and ε_t-q_ is the process error at lag *q* (MA). The parameters *Ø*_*p*_, *β*_*i*, *n*_, *ϑ*_*q*_ are estimated using least square method during fitting process [22]. Statistical analysis was performed with SPSS version 22.0.

### Ethical statement

The samples considered in this study were part of a public health program implemented by the Cameroon Ministry of Public Health. Thus, there was no need of ethical approval.

## Results

During the study period, there were 5216 naso-pharyngeal swabs collected from patients with ILI and SARI. Data regarding gender of the study population was available for 3781 participants whereas information on age distribution of the study population was available for 5136 individuals. Table 1 gives a summary of the socio-demographics of the study population.

**Table 1:** Socio-demographic characteristics of the study population.

Year	Gender N (%)		Total	Age group (years) N (%)				Total
	Female	Male		0–5	6–20	21–50	> 51	
2009	119 (44.4)	149 (55.6)	268	124 (40.1)	71 (23)	88 (28.5)	26 (8.4)	309
2010	69 (34.7)	130 (65.3)	199	148 (59.9)	42 (17)	53 (21.5)	4 (1.6)	247
2011	175 (32.6)	362 (67.4)	537	567 (81.1)	59 (8.4)	64 (9.1)	9 (1.3)	699
2012	200 (27.3)	533 (72.7)	733	872 (84.7)	91 (8.8)	57 (5.5)	10 (1)	1030
2013	208 (32)	442 (68)	650	716 (84.7)	74 (8.8)	48 (5.7)	7 (0.8)	845
2014	172 (25.6)	499 (74.4)	671	845 (84.4)	95 (9.5)	54 (5.4)	7 (0.7)	1001
2015	218 (30.2)	505 (69.8)	723	829 (82.5)	118 (11.7)	47 (4.7)	11 (1.1)	1005
Overall	1161 (30.7)	2620 (69.3)	3781	4101 (79.8)	550 (10.7)	411 (8)	74 (1.4)	5136

Among the collected specimens, 1161 (22.2%) had confirmed infections with influenza A and B virus, that is, 767 (14.7%) cases of infection with influenza A virus, 382 (7.3%) with influenza B virus and 12 (0.2%) individuals with both infections. S1 Table gives detailed information on the raw data obtained during the study. Overall, there were 362 observations with weekly data for number of influenza cases and climatic data. Table 2 summarizes the characteristics of these variables during the study period.

**Table 2:** Characteristics of study variables.

	Min	Max	Mean	Std dev	Median	IQR
**Meteorological variables (Weekly)**						
Mean temperature (°C)	21.3	27.6	24	1.1	24	23.3–24.9
Accumulated rainfall (mm)	0	179.9	28.5	31.4	19.4	1.2–43.7
Average relative humidity (%)	55.7	92.4	78.6	6.5	79.0	74.4–82.0
**Weekly counts**						
**Samples collected**	0	84	14.4	13.1	12	4–22
Overall influenza cases	0	31	3.2	4.9	1	0–4
Influenza A	0	24	2.2	4.1	0	0–2
Influenza B	0	17	1.1	2.1	0	0–1

Min = minimum, Max = maximum, Std dev = standard deviation, IQR = inter quartile range

**Fig 2:**
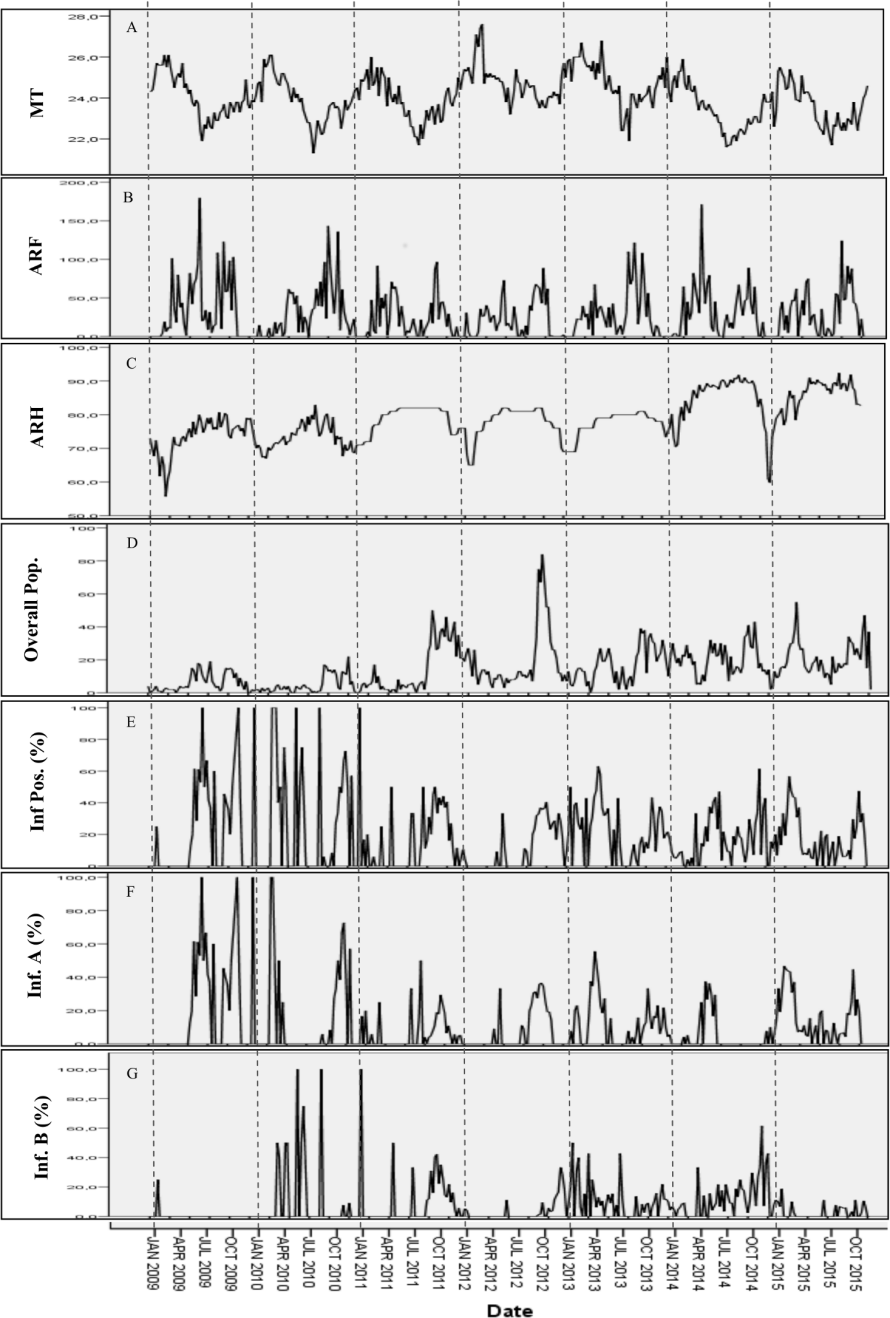
Weekly distribution of meteorological variables and influenza cases. A corresponds to mean temperature, B to accumulated rainfall, C corresponds to average relative humidity, D to overall population, E to proportion of influenza cases, F to proportion of influenza A cases, and G to proportion of influenza B cases.

Fig 2 shows the trends in influenza virus circulation and meteorological variables during the period of study. Throughout the study period, influenza activity displayed year round activity. The distribution of influenza A had a similar profile to that observed for overall influenza cases. Visually, there was no clear trend observed in the association between influenza cases and weather variables.

**Fig 3:**
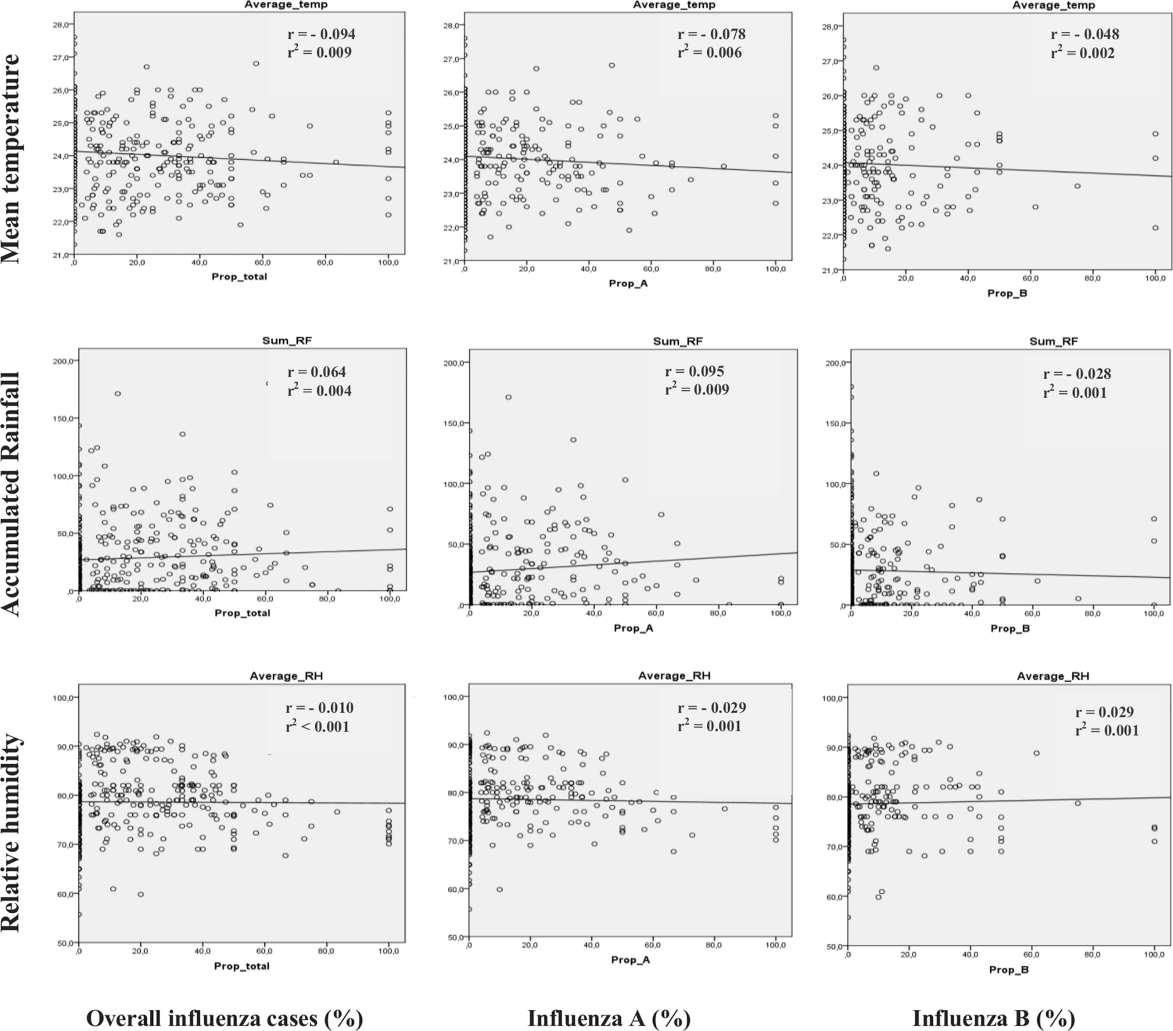
Curve estimation of relationship between influenza and weather variables.

There was no statistically significant association (P>0.05) found between influenza virus and weather variables; very weak relationships (-0.1 < r < 0.1) were observed with univariate linear regression analysis (Fig 3). Less than 1% of variations in the meteorological factors could explain the influenza activity (r^2^ < 0.01)

An Expert Modeller was used to predict on future influenza activity. This Modeller automatically found the best-fitting model for each dependent series. The Expert Modeller selected, for inclusion in ARIMA models, independent variables that have a statistically significant relationship with the dependent series. Out of the three candidate predictors fitted in the model, the Expert Modeller identified one meteorological variable that was useful for forecasting for each dependent variable. Table 3 summarizes the model performances. For overall influenza cases, the ARIMA (3,0,1) was the selected model and gave significant forecast with accumulate rainfall at lag 0 to lag 2. ARIMA model (2,0,0) was observed for influenza A with similar lags while no predictor was observed for influenza B. Accumulated rainfall was the only covariate that enabled good fit of models. The Ljung–Box (modified Box–Pierce) test indicated that model 1 for overall influenza cases was correctly specified (p-value > 0.05) and that there were no outliers with the expert modeller. Meanwhile, a p-value of below 0.05 for model 2 and 3 indicate that the residuals are not independent. Based on the stationary R^2^, 29.5% and 41.1% of the variation in the series can be explained by model 1 and 2, respectively.

**Table 3:** Summary of model performance and estimated coefficients for influenza cases.

Dep. var.	Model	Fit		Ljung–Box statistics			Meteorological variables		
		RMSE	Stat. R^2^	Statistics	d.f.	P	Variables	Est.	P
Overall Inf	ARIMA (3,0,1)	20.495	0.295	13.945	14	0.454	ARF (lag 0)	0.106	0.013
							ARF (lag 1)	-0.139	0.001
							ARF (lag 2)	-0.196	<0.001
Inf A	ARIMA (2,0,0)	15.755	0.411	30.153	16	0.017	ARF (lag 0)	0.086	0.010
							ARF (lag 1)	-0.099	0.002
							ARF (lag 2)	-0.145	<0.001
Inf B	Simple	14.858	0.401	35.315	17	0.006	-	-	-

Dep. var. = dependent variable, fit = fitting results, RMSE = Root mean square error, Stat. R^2^ = Stationary R^2^, d.f. = degree of freedom, Est. = estimate, Inf = Influenza, ARF = Accumulated Rainfall, lag = time lag of climate variables

**Fig 4:**
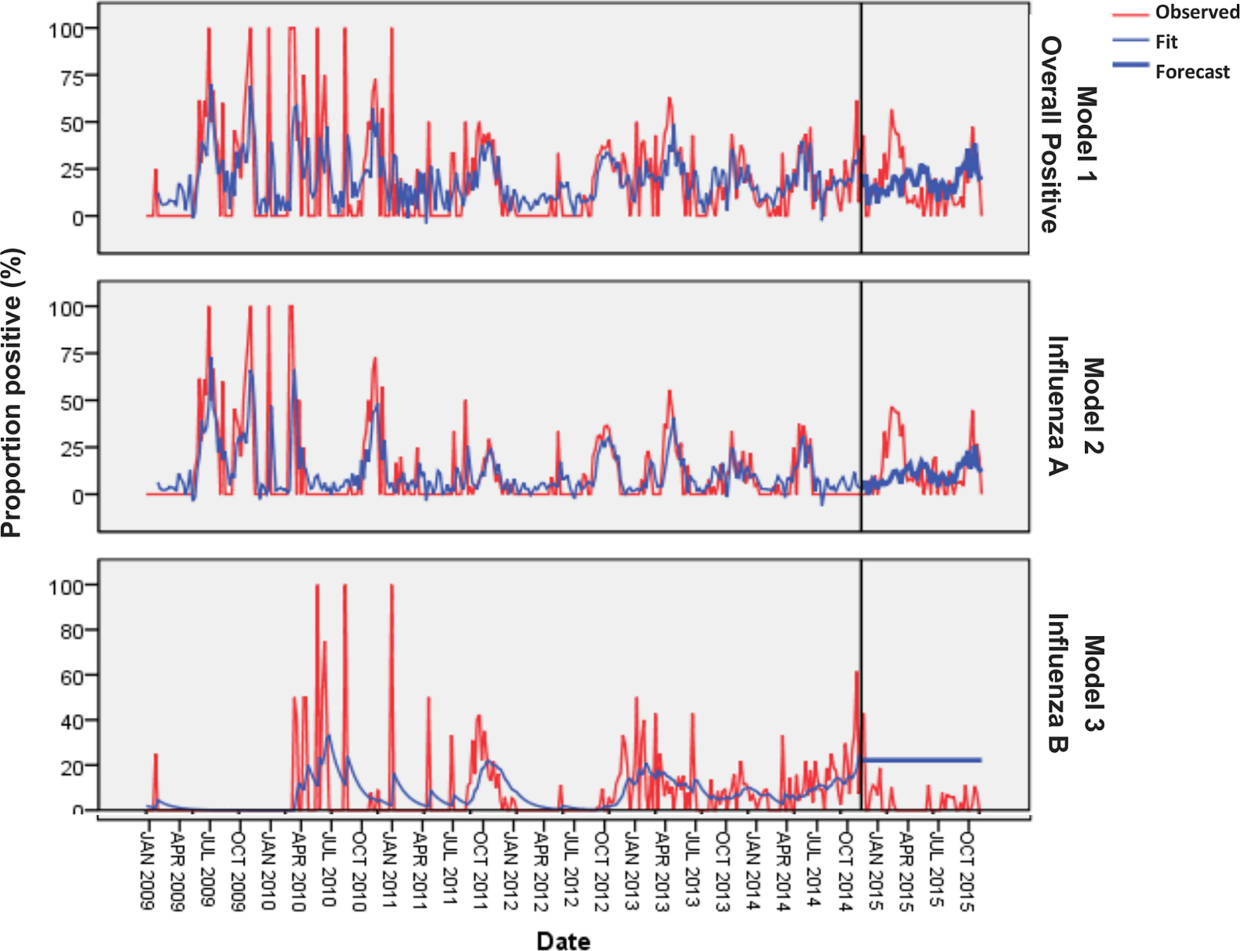
Time series profile of influenza cases with different forecasting models. Red curves are the observed data; blue curves are the modelled results; and bold-blue curves are the prospectively estimated influenza activity using actual meteorological data and ARIMA models trained with influenza data from previous years.

The graphical representation of the different models is shown in Fig 4. Model 1 (overall influenza cases) and model 2 (Influenza A cases) fitted well during the estimation period (January 2009—November 2014); however, they did not succeed to make good forecast for predictions. The estimation and prediction model for influenza A performed better than that for influenza B.

## Discussion

This study aimed to determine the relationship between influenza cases and weather variability. We found that influenza in Yaoundé is characterized by a year round activity as described by Heraud et *al*. in 2008 and 2009 respectively, who found influenza to circulate year-round or to have a seasonal peak (May through November) in Yaoundé-Cameroon [17]. These results are however different from that reported by Caini et *al*. who observed a heterogeneous pattern in several tropical countries [5].

The inverse relationship between temperature and influenza is a fact that has been long confirmed particularly in countries with temperate climates, where influenza peaks during the winter seasons [15]. Previous animal studies performed in guinea pigs showed that influenza virus is more stable in the cold with robust transmission at low temperatures due to increased virus half-life [23]. Though Cameroon is a subtropical country and has little variations in temperature across the year, a negative correlation was also observed in this study although this relationship was not significant.

Generally, data from different geographic locations show that rainfall affects influenza virus infection with increased activity depending on the region [19]. We found that the amount of rainfall is weakly related to the influenza activity. This is contradictory to reports by other studies, for example, in studies involving regions of low latitudes [15], Ivory coast [24], Thailand [25], Honduras and Nicaragua [13] where a significant correlation was observed with the number of influenza cases. A previous study reported a negative correlation between the number of influenza cases and rainfall though it was in cases of avian influenza [26]. Murray et *al*. reported that influenza A/H5N1 infections in Egypt were negatively correlated with precipitation between 2006 and 2008 [26]. The proportional correlation with rainfall can be supported by the hypothesis that rainfalls can be sufficiently intense to favour indoor gathering, thereby increasing aerosol and direct contact transmissions [24].

Relative humidity negatively correlated with the weekly influenza activity though the correlation was not significant and relatively low. Similarly, N'gattia et *al*. reported poor correlation between humidity and weekly influenza activity in Ivory Coast. It is worth noting that they observed moderately high ranges of relative humidity between 54–93% which is similar to that obtained in this study [24]. These results corroborate with past findings which reported that influenza virus circulates efficiently under dry and cold conditions [9, 17, 23].Several reports from studies performed in the tropics have however demonstrated a proportional association between influenza and humidity [14, 15, 27] which is contradictory to our findings. This can be explained by the fact that the primary mode of transmission during these humid conditions is probably by droplet and contact modes as opposed to aerosol transmission which is favoured by dry weather conditions [8].

Through the use of ARIMA models, we explored the relationship between meteorological variables and influenza activity and made forecasts with significant predictors. The Expert Modeller used in this analysis gave three models for the different viral groups (overall influenza, influenza A and influenza B). The ARIMA models did not enable predictions that were in agreement with the observed data though all models performed relatively well in the estimation period. Accumulated rainfall was the only external covariate that enabled good fit of models. Moreover, some synchronies were noted between rainfall and influenza activity as shown in Fig 2. Based on the stationary R^2^, 29.5% and 41.1% of the variation in the series could be explained by model 1 and 2, respectively. These results are the first of a kind that did not enable predictions to be made despite good fits of model [13, 25]. Association between the three weather variables and influenza may not involve a direct causal relationship and rather acted as proxies to other factors not considered in the model, such as, UV radiation, absolute humidity, air quality and wind/stability [8]. Moreover the irregular pattern observed in influenza distribution might have influenced the performance of the model. This raises more concern on the difficulty of performing timely vaccination campaigns in tropical settings. The mechanism driving influenza seasonality still remains uncertain.

Contrarily to other studies performed in developed regions, where lowering of the temperature and humidity by the air conditioning system was a commonly raised bias [13, 28], it was not an issue here. Outdoor temperature, and humidity measurements used in this study do not vary much from indoor measurements in our low income settings, where there is little availability to even electricity. Moreover, the time frame for this study was relatively long and enabled an evaluation of the different variations in influenza cases and weather parameters over time.

Although this study’s focus was to assess impacts of weather variability on influenza, there might be other environmental factors that could be associated and were not considered. For instance, absolute humidity and solar radiation have been indicated to be key environmental variables in influenza virus transmission [7, 8]. A major drawback to this study was the lack of rigorous statistical analysis such as deviance analysis that would have enabled a better understanding of the role of each weather variable in explaining the regression model. Moreover, the expert modeller used in this study is less efficient than the commonly used ARIMA model. Another drawback to this study was the lack of inclusion of other determinants capable of playing a role in the incidence of influenza such as: socio-economic elements, living environment, viral stability, host behaviour and biology.

More so, meteorological parameters were collected at a single point within the region. These point-parameters may therefore not reflect the reality of the climate conditions present in all the study sites due to possible variations in these factors from one location to another.

The fact that this study was performed in only one geographic location did not enable comparisons to be made with other regions within the same country in order to evaluate variations obtained with changes in climate and socio-economic factors.

## Conclusion

This study laid more emphasis on the fact that influenza in Cameroon is characterized by year-round activity. The exploited meteorological parameters did not give significant forecasts on expected influenza cases during the prediction period probably due to several other environmental factors not included in the model that might have been implicated in the circulation of this virus as well as intrinsic factors specific to the individual or socio-economic elements. Future studies taking into consideration these factors are warranted.

## Supporting information

S1 Table Complete study data. MT = Mean temperature, ARF = Accumulated Rainfall, ARH = Average Relative Humidity, Inf. = Influenza.
(PDF)
