# Supplementary material for: Associations between meteorological parameters and influenza activity in a subtropical country: Case of five sentinel sites in Yaoundé-Cameroon
Source: PLoS One. 2017 Oct 31;12(10):e0186914. doi: 10.1371/journal.pone.0186914 (PMC5663393; doi:10.1371/journal.pone.0186914)
Supplement: S1 Table — MT = Mean temperature, ARF = Accumulated Rainfall, ARH = Average Relative Humidity, Inf. = Influenza. (PDF) [file pone.0186914.s001.pdf]

Year	Week	MT	ARF	ARH	Samples tested	Samples +ve	Inf. A +ve	Inf. B +ve	Proportion +ve (%)
2009	w1	24.3	0	73	4	0	0	0	0
2009	w2	24.4	0	70.1	0	0	0	0	0
2009	w3	25	0	67.7	1	0	0	0	0
2009	w4	25.7	0	72.4	2	0	0	0	0
2009	w5	25.6	0	68.1	4	1	0	1	25
2009	w6	25.6	0	61.7	1	0	0	0	0
2009	w7	25.6	4	67.6	1	0	0	0	0
2009	w8	26.1	19.1	64.9	0	0	0	0	0
2009	w9	25.5	6.2	55.7	2	0	0	0	0
2009	w10	26.1	11.4	60.9	3	0	0	0	0
2009	w11	25.5	10.8	63.3	2	0	0	0	0
2009	w12	25	101.4	68.7	2	0	0	0	0
2009	w13	24.5	35.9	73.4	2	0	0	0	0
2009	w14	25	18.1	71	2	0	0	0	0
2009	w15	25.2	80	71	0	0	0	0	0
2009	w16	25	37.7	70.6	2	0	0	0	0
2009	w17	25.7	43.5	72.9	4	0	0	0	0
2009	w18	24.5	15.4	73.6	3	0	0	0	0
2009	w19	24.7	1.2	72	3	0	0	0	0
2009	w20	24.2	43.7	74.4	4	0	0	0	0
2009	w21	24.4	82.1	75.9	3	0	0	0	0
2009	w22	23.8	42	73.3	8	1	1	0	13
2009	w23	23.6	68.1	77.9	15	3	3	0	20
2009	w24	23.9	74.3	74.1	13	8	8	0	62
2009	w25	23.7	94.9	76.7	7	2	2	0	29
2009	w26	22.4	179.9	80	18	11	11	0	61
2009	w27	21.9	20	77.9	17	9	9	0	53
2009	w28	22.7	21.5	74.7	11	11	11	0	100
2009	w29	22.5	34	76.1	8	4	4	0	50
2009	w30	23.1	8.7	79	6	4	4	0	67
2009	w31	22.6	31.5	77.3	12	5	5	0	42
2009	w32	22.5	4.5	79.1	19	7	7	0	37
2009	w33	23.3	13.7	75.6	7	0	0	0	0
2009	w34	22.9	15.6	76.3	5	3	3	0	60
2009	w35	22.9	108.6	75.6	3	0	0	0	0
2009	w36	23.3	57.3	80.7	4	0	0	0	0
2009	w37	23.4	9.8	76.3	1	0	0	0	0
2009	w38	23.7	123	78.1	3	0	0	0	0
2009	w39	23.1	57.5	80.4	11	5	5	0	46
2009	w40	23	58.7	79.9	15	6	6	0	40
2009	w41	23.8	98.2	73.9	14	5	5	0	36
2009	w42	23.6	33.8	73	15	3	3	0	20
2009	w43	23.3	102.9	75.7	14	7	7	0	50

2009	w44	23.8	50.5	76	9	6	6	0	67
2009	w45	23.8	0	76.6	12	10	10	0	83
2009	w46	23.3	0	76.9	4	4	4	0	100
2009	w47	23.8	0	75.1	9	4	4	0	44
2009	w48	23.9	0	76	5	0	0	0	0
2009	w49	24.9	0	73.6	2	0	0	0	0
2009	w50	23.7	0	79	7	0	0	0	0
2009	w51	23.5	0	78.6	0	0	0	0	0
2009	w52	23.9	0	79	0	0	0	0	0
2010	w1	23.8	0	75.7	2	0	0	0	0
2010	w2	24.1	0	72.6	1	1	1	0	100
2010	w3	24.7	0	70	2	0	0	0	0
2010	w4	24.8	14.5	71	2	0	0	0	0
2010	w5	23.8	0	70.1	0	0	0	0	0
2010	w6	25.9	0	67.3	3	0	0	0	0
2010	w7	25.5	0	67.6	1	0	0	0	0
2010	w8	25.7	0	67	2	0	0	0	0
2010	w9	26.1	10.8	70.3	3	0	0	0	0
2010	w10	26.1	0	69.4	0	0	0	0	0
2010	w11	25.3	0	70.1	1	1	1	0	100
2010	w12	25	18.5	71.3	1	1	1	0	100
2010	w13	24.7	3.7	71.7	4	4	2	2	100
2010	w14	24.6	13.7	71.4	5	2	0	2	40
2010	w15	25.2	18	72.1	2	1	1	0	50
2010	w16	25.2	0	72.7	2	0	0	0	0
2010	w17	24.9	5.1	73.7	4	3	1	2	75
2010	w18	24.7	40.7	71	2	1	0	1	50
2010	w19	24.2	61.4	72.1	4	0	0	0	0
2010	w20	23.8	55.4	72.1	1	0	0	0	0
2010	w21	24.5	57.5	74.7	0	0	0	0	0
2010	w22	24	41.7	74.1	0	0	0	0	0
2010	w23	24.2	52.8	73.9	4	4	0	4	100
2010	w24	23.8	5.2	76	3	0	0	0	0
2010	w25	24	39.8	75.9	2	1	0	1	50
2010	w26	23.4	5.3	78.7	4	3	0	3	75
2010	w27	23.1	25.3	77.6	5	2	0	2	40
2010	w28	22.8	18.2	75	3	0	0	0	0
2010	w29	22.8	0	79.4	3	0	0	0	0
2010	w30	22.2	24.8	77	2	0	0	0	0
2010	w31	21.3	35	79.9	0	0	0	0	0
2010	w32	22	36.5	78.6	1	0	0	0	0
2010	w33	22.9	61.8	82.9	0	0	0	0	0
2010	w34	22.6	39.5	77.3	1	0	0	0	0
2010	w35	22.2	71	73.6	1	1	0	1	100
2010	w36	22.3	38.8	74.6	5	0	0	0	0

2010	w37	22.8	96.5	77.3	17	1	1	0	6
2010	w38	23	23.1	79.6	16	0	0	0	0
2010	w39	23.6	143.4	80.3	13	0	0	0	0
2010	w40	23.7	90.6	76.1	14	0	0	0	0
2010	w41	23.8	59.3	74.6	12	1	1	0	8
2010	w42	23.5	29.6	79.3	14	0	0	0	0
2010	w43	23.6	65.7	71.1	11	3	3	0	27
2010	w44	23.4	136	73.7	3	1	1	0	33
2010	w45	22.5	7.8	72.7	2	1	1	0	50
2010	w46	23.1	61	73.4	13	6	5	1	46
2010	w47	23.9	32.7	67.7	12	8	8	0	67
2010	w48	23.4	20.2	71.1	11	8	8	0	73
2010	w49	23.6	14.3	69.3	22	11	9	2	50
2010	w50	23.8	1.8	69.9	5	0	0	0	0
2010	w51	24.1	13.3	72.3	7	4	4	0	57
2010	w52	24.4	22.4	68.9	1	0	0	0	0
2011	w1	24.5	0	68.6	0	0	0	0	0
2011	w2	23.9	0	71	0	0	0	0	0
2011	w3	24.9	0	71	1	1	0	1	100
2011	w4	24.8	0	71	5	0	0	0	0
2011	w5	25	0	71	6	1	1	0	17
2011	w6	25.4	0	71.9	3	0	0	0	0
2011	w7	24.4	6.2	72	5	1	1	0	20
2011	w8	26	0	72	5	0	0	0	0
2011	w9	24.6	47.3	72	7	0	0	0	0
2011	w10	24.8	7.6	76.3	17	1	1	0	6
2011	w11	25.5	4	77	6	0	0	0	0
2011	w12	24.1	91.7	77	10	0	0	0	0
2011	w13	25.5	12.7	77	2	0	0	0	0
2011	w14	25.2	50.1	78.3	4	1	1	0	25
2011	w15	25	36.4	80	2	0	0	0	0
2011	w16	23.6	54.9	80	2	0	0	0	0
2011	w17	25	1.4	80	2	0	0	0	0
2011	w18	24.1	19.6	80.1	0	0	0	0	0
2011	w19	23.8	70.8	81	2	1	0	1	50
2011	w20	24.2	61.7	81	2	0	0	0	0
2011	w21	23.7	64.8	81	0	0	0	0	0
2011	w22	24.6	51.4	81	8	0	0	0	0
2011	w23	23.7	12.8	81.7	2	0	0	0	0
2011	w24	23.8	39.3	82	5	0	0	0	0
2011	w25	23.1	23.3	82	4	0	0	0	0
2011	w26	23.1	0.9	82	3	0	0	0	0
2011	w27	23.4	7.7	82	5	0	0	0	0
2011	w28	22.6	4.7	82	5	0	0	0	0
2011	w29	22.6	11.4	82	6	2	0	2	33

2011	w30	22.1	23	82	3	1	1	0	33
2011	w31	22.1	11	82	0	0	0	0	0
2011	w32	21.7	0	82	4	0	0	0	0
2011	w33	22.7	0	82	6	0	0	0	0
2011	w34	22	21.6	82	7	1	1	0	14
2011	w35	22.7	0	82	2	1	1	0	50
2011	w36	23.4	9.3	82	9	1	0	1	11
2011	w37	22.9	14.7	82	25	4	1	3	16
2011	w38	23.4	20.5	82	29	9	0	9	31
2011	w39	23.6	43.6	82	50	10	2	8	20
2011	w40	22.7	17	82	42	19	2	17	45
2011	w41	23.3	86.9	82	26	13	2	11	50
2011	w42	23.7	96.6	82	27	9	3	6	33
2011	w43	22.8	22	82	37	16	7	13	43
2011	w44	22.9	44	82	39	15	7	10	39
2011	w45	23.8	45.1	81.1	34	15	10	5	44
2011	w46	24.3	33.8	81	46	17	11	6	37
2011	w47	24.5	12.6	81	32	13	6	7	41
2011	w48	23.8	29.3	81	31	5	2	3	16
2011	w49	23.6	10	77	37	10	4	6	27
2011	w50	24	0	74	43	4	2	2	9
2011	w51	24.2	0	74	22	0	0	0	0
2011	w52	25	12.8	74	35	4	2	2	11
2012	w1	24.4	0	74.3	21	0	0	0	0
2012	w2	25	0	76	21	1	1	0	5
2012	w3	25.4	0	76	19	2	1	1	11
2012	w4	25.3	0	76	24	1	0	1	4
2012	w5	25.5	30.8	76	27	0	0	0	0
2012	w6	25.1	5.7	68.1	19	0	0	0	0
2012	w7	24.7	0	65	10	0	0	0	0
2012	w8	25.9	0	65	26	0	0	0	0
2012	w9	27.1	0	65	11	0	0	0	0
2012	w10	26.5	0	70.7	5	0	0	0	0
2012	w11	27.4	5.7	75	11	0	0	0	0
2012	w12	27.6	35.7	75	14	0	0	0	0
2012	w13	24.7	25.9	75	14	0	0	0	0
2012	w14	25.2	26.6	75.4	11	0	0	0	0
2012	w15	25	40.2	78	13	0	0	0	0
2012	w16	25.2	33.8	78	6	0	0	0	0
2012	w17	25	18	78	3	0	0	0	0
2012	w18	25.1	24.7	78	9	0	0	0	0
2012	w19	24.9	27.5	79.7	8	0	0	0	0
2012	w20	25	7.1	80	11	1	1	0	9
2012	w21	24.8	15.6	80	11	0	0	0	0
2012	w22	24.8	20	80	7	0	0	0	0

2012	w23	24.4	56	80.9	3	1	1	0	33
2012	w24	23.8	72.8	82	9	1	0	1	11
2012	w25	24.5	13.7	82	6	0	0	0	0
2012	w26	23.2	17.3	82	8	0	0	0	0
2012	w27	23.7	12.7	81.9	8	0	0	0	0
2012	w28	24.3	7.8	81	8	0	0	0	0
2012	w29	25.4	38.6	81	9	0	0	0	0
2012	w30	24.8	21.8	81	13	0	0	0	0
2012	w31	24.7	0	81	13	0	0	0	0
2012	w32	24	0	81	10	0	0	0	0
2012	w33	24.3	8.8	81	11	0	0	0	0
2012	w34	24.9	0	81	9	1	1	0	11
2012	w35	24.7	13.9	81	11	1	1	0	9
2012	w36	24.5	1.8	81	14	0	0	0	0
2012	w37	24.6	40.1	81	7	0	0	0	0
2012	w38	24.6	33.5	81	20	4	4	0	20
2012	w39	24	56.2	81	34	8	8	0	24
2012	w40	23.8	67.7	81	75	24	23	1	32
2012	w41	23.5	65.2	82	67	22	21	1	33
2012	w42	23.9	62.4	82	84	31	23	8	37
2012	w43	23.6	43.1	82	67	24	24	0	36
2012	w44	23.5	88.7	82	52	19	19	0	37
2012	w45	24	40.9	79.7	52	21	18	3	40
2012	w46	24.1	61.8	78	31	9	8	1	29
2012	w47	24	0	78	26	6	5	1	23
2012	w48	24.3	1	78	26	7	5	3	27
2012	w49	24.1	0	77.4	21	6	3	3	29
2012	w50	24.2	0	76	12	2	0	2	17
2012	w51	23.7	0	76	12	4	0	4	33
2012	w52	25.1	0	76	7	2	0	2	29
2012	w53	25.7	0	76	11	2	0	2	18
2013	w1	25	1.8	70	7	0	0	0	0
2013	w2	25.8	0	69	13	1	0	1	8
2013	w3	25.9	0	69	5	1	0	1	20
2013	w4	24.8	0	69	4	2	0	2	50
2013	w5	26	0	69	7	0	0	0	0
2013	w6	26	0	69	13	5	1	4	39
2013	w7	26	12.3	69	15	6	0	6	40
2013	w8	26	22.7	69	15	4	3	1	27
2013	w9	26.7	22.3	72	13	3	3	0	23
2013	w10	26	21.5	76	13	4	2	2	31
2013	w11	25.7	39.5	76	4	0	0	0	0
2013	w12	25.5	25.1	76	7	3	0	3	43
2013	w13	25.6	29.3	76	0	0	0	0	0
2013	w14	25.3	9.1	76	4	1	0	1	25

2013	w15	25.8	46.2	76	13	4	2	2	31
2013	w16	24.9	1.3	76	16	7	6	1	44
2013	w17	25.7	67.5	76	23	11	8	3	48
2013	w18	25.2	23.7	78.1	27	17	15	2	63
2013	w19	26.8	36.2	79	19	11	9	2	58
2013	w20	25.7	39.5	79	19	7	7	0	37
2013	w21	24.4	30.5	79	22	7	5	2	32
2013	w22	24.7	20.8	79	27	9	5	4	33
2013	w23	25.1	11.6	79	22	8	6	2	36
2013	w24	24.4	37.7	79	13	2	0	2	15
2013	w25	24.2	12	79	6	0	0	0	0
2013	w26	24.4	42.6	79	13	3	2	1	23
2013	w27	24.2	22.4	80	2	0	0	0	0
2013	w28	24.6	2.1	80	7	3	0	3	43
2013	w29	22.4	9.2	80	16	2	0	2	13
2013	w30	22.4	14.4	80	6	0	0	0	0
2013	w31	23	2.4	80	2	0	0	0	0
2013	w32	23.5	15.2	80	8	0	0	0	0
2013	w33	21.9	31.1	80	10	0	0	0	0
2013	w34	24.2	110	80	4	0	0	0	0
2013	w35	24.2	67.7	80	13	1	1	0	8
2013	w36	23.8	73.4	80	22	3	0	3	14
2013	w37	23.5	121.6	80	25	1	1	0	4
2013	w38	24.2	74.6	80	39	0	0	0	0
2013	w39	23.8	15.7	80	36	5	2	3	14
2013	w40	24	42.9	80.9	38	7	6	1	18
2013	w41	24.1	108.3	81	12	1	0	1	8
2013	w42	24.2	73.7	81	33	5	2	3	15
2013	w43	24.6	25.3	81	36	4	4	0	11
2013	w44	23.9	56.1	80.1	31	6	4	2	19
2013	w45	23.5	20.3	79	30	13	10	3	43
2013	w46	24.4	6	79	19	6	3	3	32
2013	w47	24.7	0.2	79	22	2	2	0	9
2013	w48	25.1	14.5	78.9	24	7	4	3	29
2013	w49	24.9	11	78	32	12	5	7	38
2013	w50	25.5	0	78	26	9	6	3	35
2013	w51	24.5	0	78	17	3	1	2	18
2013	w52	26	0	78	10	2	1	1	20
2014	w1	24.3	0.4	75.9	23	5	5	1	22
2014	w2	23.8	0	73.3	30	5	3	2	17
2014	w3	24.8	0	74.9	23	2	1	1	9
2014	w4	25.1	0	76.3	18	1	1	0	6
2014	w5	24.5	3.6	80.2	21	1	0	1	5
2014	w6	25.3	3.8	73.3	16	1	0	1	6
2014	w7	25.3	0	70.4	23	2	0	2	9

2014	w8	25.9	0	71.1	22	2	0	2	9
2014	w9	24.7	27.4	79	29	0	0	0	0
2014	w10	24.4	64.8	82.3	21	0	0	0	0
2014	w11	25.1	1.4	78.2	24	1	1	0	4
2014	w12	24.4	44.8	84.1	17	0	0	0	0
2014	w13	24.3	21.8	82.3	19	1	1	0	5
2014	w14	24.6	31	80.5	5	0	0	0	0
2014	w15	23.9	82	83.5	6	2	0	2	33
2014	w16	23.4	62.4	87.3	5	0	0	0	0
2014	w17	23.8	46.6	86.1	11	0	0	0	0
2014	w18	23.9	30.2	84.9	7	1	0	1	14
2014	w19	23.5	171.2	88.9	16	2	2	0	13
2014	w20	23.4	76.4	87.3	24	6	6	0	25
2014	w21	23.7	42.4	86.8	32	5	0	5	16
2014	w22	23.5	70.2	88	24	9	9	1	38
2014	w23	23.3	79.4	89.3	30	10	8	2	33
2014	w24	23.1	12.4	88.5	22	9	8	2	41
2014	w25	23.2	6.4	88.1	23	10	7	5	44
2014	w26	22.8	45.2	88.9	30	7	5	2	23
2014	w27	22.8	9.6	88.4	17	8	5	3	47
2014	w28	22.1	0.2	88.3	29	1	0	1	3
2014	w29	22.3	0.2	87.5	23	5	0	5	22
2014	w30	21.6	0.2	89.8	7	1	0	1	14
2014	w31	21.7	19.2	90.4	22	2	0	2	9
2014	w32	21.7	28.4	89.7	11	1	0	1	9
2014	w33	22.1	11.2	88.1	13	0	0	0	0
2014	w34	22.2	18	88.9	16	3	0	3	19
2014	w35	21.9	10	90.4	15	2	0	2	13
2014	w36	22.3	31.6	89.4	12	3	0	3	25
2014	w37	22.5	51	90.9	16	3	0	3	19
2014	w38	22.4	67.2	90.6	34	6	0	6	18
2014	w39	22.2	40.6	91.8	35	3	0	3	9
2014	w40	22.5	27	89.7	41	1	0	1	2
2014	w41	23	46.4	89.5	28	3	0	3	11
2014	w42	22.7	48.4	91	27	8	0	8	30
2014	w43	22.8	89	90.1	43	9	0	9	21
2014	w44	23.1	43	89.4	28	2	0	2	7
2014	w45	23.1	29	89.4	15	4	0	4	27
2014	w46	22.8	64.4	90.1	18	6	0	6	33
2014	w47	22.8	19.8	88.8	13	8	0	8	62
2014	w48	23.7	0	86.2	14	1	0	1	7
2014	w49	24.2	0	82.3	11	4	0	4	36
2014	w50	23.8	18.6	84.7	14	6	0	6	43
2014	w51	23.8	0.4	82.2	2	0	0	0	0
2014	w52	23.8	0	76.7	4	0	0	0	0

2015	w1	24.3	0	74.6	13	2	1	1	15
2015	w2	22.6	0	60.9	9	1	0	1	11
2015	w3	23.3	0	59.8	10	2	1	1	20
2015	w4	25.3	0	73.3	15	1	0	1	7
2015	w5	25.5	0	75.8	16	4	1	3	25
2015	w6	25.2	1	78.8	15	2	2	0	13
2015	w7	25	35	79.8	18	6	6	0	33
2015	w8	24.4	44.6	80.5	26	5	5	0	19
2015	w9	24.5	8.2	81.9	30	10	10	0	33
2015	w10	25.4	27.2	76.9	30	17	14	3	57
2015	w11	25	4.4	78.6	38	18	17	1	47
2015	w12	24.7	37	84	55	24	24	0	44
2015	w13	23.9	47.2	87.1	30	13	13	0	43
2015	w14	23.9	46	84.9	26	8	8	0	31
2015	w15	23.8	61.8	85.8	27	10	10	0	37
2015	w16	25.1	0.2	78.4	16	2	2	0	13
2015	w17	24.2	26.6	82.2	14	1	1	0	7
2015	w18	24.3	46.8	84.3	12	1	1	0	8
2015	w19	24.3	27.4	84.7	14	1	1	0	7
2015	w20	24.1	70.8	87.2	18	2	2	0	11
2015	w21	23.8	74.8	86.5	19	1	1	0	5
2015	w22	23.9	14.6	87.8	22	1	1	0	5
2015	w23	22.9	25.8	91	13	2	2	0	15
2015	w24	22.8	28.8	89.1	20	0	0	0	0
2015	w25	22.5	9.8	89.8	16	1	1	0	6
2015	w26	22.4	0.2	89	9	2	1	1	22
2015	w27	22.2	1.2	90.4	12	0	0	0	0
2015	w28	23.4	35.6	89.5	11	2	2	0	18
2015	w29	22.6	2.6	89.5	5	1	1	0	20
2015	w30	22.1	0	88.6	22	0	0	0	0
2015	w31	21.7	11	89.1	12	1	1	0	8
2015	w32	22.7	7.4	87.2	13	2	1	1	15
2015	w33	22.6	0	87.8	18	0	0	0	0
2015	w34	23.3	3.8	86.1	15	1	0	1	7
2015	w35	22.7	55.6	89.2	16	3	2	1	19
2015	w36	22.4	54	89.6	18	2	1	1	11
2015	w37	22.8	16.2	88.5	17	1	0	1	6
2015	w38	22.4	124.2	92.4	34	2	2	0	6
2015	w39	23	46.8	87.5	31	2	2	0	7
2015	w40	22.9	48	89.3	30	3	2	1	10
2015	w41	22.7	91.4	89.3	23	1	1	0	4
2015	w42	23.8	68.4	87	27	8	5	3	30
2015	w43	23.1	88	89.3	23	4	4	0	17
2015	w44	22.4	43.8	91.9	20	5	5	0	25
2015	w45	23.1	42.6	88	38	18	17	1	47

2015	w46	23.5	30	87.2	47	13	8	5	28
2015	w47	24	1	83	15	5	4	1	33
2015	w48	24.2	22.6	83.1	37	7	7	0	19
2015	w49	24.6	0	82.7	2	0	0	0	0
